# AGE-ASSOCIATED INCREASE IN KYNURENINE INHIBITS AUTOPHAGY AND PROMOTES SENESCENCE IN BONE MARROW STEM CELLS

**DOI:** 10.1093/geroni/igz038.3469

**Published:** 2019-11-08

**Authors:** Dmitry Kondrikov, Ahmed Elmansi, Xing-ming Shi, Sadanand Fulzele, Meghan mcGee-Lawrence, Mark Hamrick, Carlos Isales, William D Hill

**Affiliations:** 1 Medical University of South Carolina, Charleston, South Carolina, United States; 2 Augusta University, Augusta, Georgia, United States; 3 Department of Orthopaedic Surgery, Medical College of Georgia, Augusta University, Augusta, Georgia, United States

## Abstract

Aging is characterized by progressive decline of tissue functionality and age-related accumulation of cellular and molecular damage leading to multiple pathological conditions including osteoporosis and increased fracture rates. Bone marrow mesenchymal stem cells (BMSCs) play an essential role in bone development and regeneration with their ability to undergo differentiation into osteogenic, chondrogenic, myogenic, and adipogenic cell lines cell lines. Proliferation rate of MSC is declined with ages leading to misbalance between bone resorption and osteogenesis. A recently identified age-related change in bone and bone marrow is an accumulation of tryptophan metabolite, kynurenine (KYN), catalyzed by indoleamine-2,3-dioxygenase (IDO) or free-radical oxidation. We previously reported that KYN suppresses autophagy in BMSC. We now investigated the effect of KYN on BMSC cellular function. In vitro treatment of murine BMSC isolated from 18 month old mice with kynurenine disrupted autophagy suppressing autophagic flux. KYN treatment also induces senescence in BMSC marked by increase in SA-beta-galactosidase activity as well as, increased expression of senescence marker p21. Inhibition of Aryl Hydrocarbon Receptor (AhR) by AhR inhibitors significantly reduced β-galactosidase activity increase and blocked p21 expression elevation suggesting that KYN induces senescence in BMSC through the AhR pathway. Interestingly, KYN treatment failed to up-regulate beta-gal activity in BMSC isolated from 6 month-old mice suggesting that KYN induction of senescence maybe potentiated with aging. Together those data support the idea that KYN shifts the homeostatic balance of BMSC during prolonged stress or in aging through downregulating survival autophagic pathway in favor of driving BMSCs to senescence.

